# Global genetic diversity and geographical distribution of *Bemisia tabaci* and its bacterial endosymbionts

**DOI:** 10.1371/journal.pone.0213946

**Published:** 2019-03-19

**Authors:** Surapathrudu Kanakala, Murad Ghanim

**Affiliations:** Department of Entomology, Agricultural Research Organization—the Volcani Center, Rishon LeZion, Israel; Chinese Academy of Agricultural Sciences, CHINA

## Abstract

*Bemisia tabaci* is one of the most threatening pests in agriculture, causing significant losses to many important crops on a global scale. The dramatic increase and availability of sequence data for *B*. *tabaci* species complex and its bacterial endosymbionts is critical for developing emerging sustainable pest management strategies which are based on pinpointing the global diversity of this important pest and its bacterial endosymbionts. To unravel the global genetic diversity of *B*. *tabaci* species complex focusing on its associated endosymbionts, along with Israeli whitefly populations collected in this study, we combined available sequences in databases, resulting in a total of 4,253 mitochondrial cytochrome oxidase I (mtCOI) sequences from 82 countries and 1,226 16S/23S rRNA endosymbiont sequences from 32 countries that were analyzed. Using Bayesian phylogenetic analysis, we identified two new *B*. *tabaci* groups within the species complex and described the global distribution of endosymbionts within this complex. Our analyses revealed complex divergence of the different endosymbiont sequences within the species complex, with overall one *Hamiltonella*, two *Porteria* (P1 and P2), two *Arsenophonus* (A1 and A2), two *Wolbachia* (super-groups O and B), four *Cardinium* (C1-C4) and three *Rickettsia* (R1-R3) groups were identified. Our comprehensive analysis provides an updated important resource for this globally important pest and its secondary symbionts, which have been a major subject for research in last three decades.

## Introduction

The whitefly *Bemisia tabaci* (Gennadius) (Hemipetra: Aleyrodidae), is one of the most economically and agriculturally important insect pests worldwide. This pest causes serious economic damage by direct feeding and by transmitting plant viruses that belong to several virus families including *Begomovirus*, *Crinivirus*, *Carlavirus*, *Torradovirus* and *Ipomovirus* [1,2]. During the past two decades, begomoviruses and *B*. *tabaci* are considered the most economically important virus-vector complexes, threatening major vegetable, field and fiber crops on a worldwide scale especially in developing countries where stable food availability is scarce. *B*. *tabaci* was the first major global invasive pest in the Middle East during the 1980’s, and rapidly spread to other regions mainly via human activities and international trade.

The diversity of *B*. *tabaci* sibling species complex has been extensively studied and several biological and molecular characteristics such as plant host preference, fecundity, ability to transmit begomoviruses, dispersal, resistance to insecticide and mitochondrial cytochrome oxidase I (mtCOI) DNA sequences, have been used to compare the different genetic groups [3–10]. Recent molecular markers were used in phylogenetic analyses to identify the different worldwide populations of *B*. *tabaci* [7]. Sequencing a 657 bp portion of mtCOI helped identifying new species of *B*. *tabaci* and established the new term "cryptic species complex" [7,11]. On the basis of 3.5% pairwise divergence in mtCOI sequences within *B*. *tabaci* species, 42 distinct species have been reported: Africa, Asia I, Asia I-India, Asia II 1–12, Asia III, Asia IV, Asia V, Australia, Australia/Indonesia, China 1–5, Indian Ocean, Ru, Middle East Asia Minor I-II (MEAM), Mediterranean (MED), MEAM K, New World 1–2, Japan 1–2, Uganda, Italy 1, and Sub Saharan Africa 1–5 [7,10–14]. Recently, it was suggested that 4% genetic divergence is more realistic than the 3.5% in the identification of new species within this complex [15]. Although current studies provide a good understanding of this complex, the diversity of species within the complex is expected to be higher and more analyses are warranted for the discovery of such new species.

Similar to many other arthropods, *B*. *tabaci* species complex carries a primary endosymbiotic bacterium called *Candidatus Portiera aleyrodidarum* [16,17], which is fixed in populations and confined to bacteriocyte cells in all whitefly individuals. This bacterium is essential for host survival, development and has a long co-evolutionary history with all members of the subfamily Aleyrodidae [16,18,19]. In addition to the primary endosymbiont, seven different secondary endosymbionts namely *Rickettsia* [20], *Wolbachia* [21,22], *Hamiltonella* and *Arsenophonus* [16,17], *Cardinium* [23], *Fritschea* [24], and *Hemipteriphilus* [25] have been reported from *B*. *tabaci* populations around the world.

Except for *Portiera*, secondary symbionts differ in their 1) composition/infection frequencies in *B*. *tabaci* populations [16,26–32] 2) Spacial localization phenotypes/patterns in developmental and adult stages [26,30,31] and 3) Horizontal/vertical transfer [33–36] within and between populations. Several of these secondary endosymbionts interfere with host physiology, ecology and reproduction [37–41], rapid evolutionary shifts [42], thermotolerance [43], resistance to insecticides [44], host fitness [45], defense against pathogens [46] and influence virus transmission abilities by members of this species complex [33,47–49]. These studies suggest that identifying the infection status of endosymbionts in *B*. *tabaci* populations is highly relevant for understanding their association with *B*. *tabaci* genetic groups.

Several *B*. *tabaci* species populations around the world were surveyed for infection with endosymbionts and clear variations in the infection by the different endosymbionts within the *Bemisia* genetic groups were repeatedly observed [25–32,40]. For example, in populations tested from China *Wolbachia*, *Rickettsia*, *Arsenophonus*, *Hamiltonella* and *Cardinium* were detected in both MEAM1 and MED populations [50]. Another study from China detected *Hamiltonella* in both MEAM1 and MED, *Rickettsia* in Asia II 3, Asia II 7, China 1 and MEAM1. None of the MEAM1 and MED populations from China were infected with *Wolbachia*, *Cardinium* and *Arsenophonus* [25]. *Arsenophonus*, *Cardinium*, *Rickettsia* and *Wolbachia* were detected in native whiteflies of Africa [32], China [25] and India [51] but not *Hamiltonella* and *Fritschea*. Similarly, individual infection status of secondary symbionts from Tunisia identified *Hamiltonella* and *Rickettsia* from MEAM1 and only *Hamiltonella* from Q1 [29]. A recent study by Gorsane *et al*. [52] hypothesized that the presence of *Cardinium* in MEAM1 and *Cardinium*, *Fritschea* and *Wolbachia* in MED may explain the differences in infection status possibly due to plant hosts, site to site variations, year to year surveys, low titer of endosymbiont, influence of chemical insecticides, and technical detection problems like PCR, which could result in false negatives.

In this study, we sequenced part of mtCOI and 16S/23S rRNA genes from whitefly populations recently collected in Israel to identify and update the status of *Bemisia* genetic groups and their infection with bacterial endosymbionts, and to test whether the situation has changed since the last study conducted in this regard by Chiel *et al*. [40]. To put this study in a broader context, the sequences collected from Israel were combined with sequences available in databases, to identify *B*. *tabaci* worldwide species and their associated endosymbiont distribution. Among few molecular techniques, mtCOI and 16S/23S rRNA molecular markers are widely used to improve the robustness of the phylogenetic relationships among *B*. *tabaci* species identification, and genetically diverse distinct groups/strains of endosymbionts within the genetic groups of *Bemisia*. Our objectives were thus to determine 1) global diversity and geographic distribution of *B*. *tabaci* species complex and endosymbionts infection within *Bemisia* genetic groups worldwide and 2) provide baseline information on genetically diverse distinct groups/strains of endosymbionts within *Bemisia* genetic groups across the world, using Bayesian phylogenetic analysis.

## Materials and methods

### *B*. *tabaci* genotype identification

*B*. *tabaci* populations were collected as adults using a hand-held aspirator from several agricultural fields in Israel. After collection, the insects were transferred to glass bottle containing cotton leaves. About twenty whitefly adults were used to test the purity of the B/Q biotype population which was confirmed by PCR with Bem 23 Forward- CGGAGCTTGCGCCTTAGTC and Reverse- CGGCTTTATCATAGCTCTCGT specific microsattelite primers, that give expected PCR product of 200 bp for B and 400 bp for Q [53]. Whitefly species identity confirmation was performed using the primers C1-J-2195 (5’-TTGATTTTTTGGTCATCCAGAAGT-3’) and L2-N-3014 (5’-TCCAATGCACTAATCTGCCATATTA-3’) that amplify a fragment from the mitochondrial cytochrome oxidase I gene (mtCOI) [8]. Each species was maintained on cotton seedlings (*Gossypium hirsutum*L. cv. Acala) inside insect-proof cages and growth rooms under standard conditions of 25 ± 2°C, 60% relative humidity and a 14 h light/10 h dark photoperiod for further studies.

### Detection of endosymbionts

The same DNA originated from each individual was used for screening of all primary (*Portiera*) and secondary symbionts (*Hamiltonella*, *Rickettsia*, *Wolbachia*, *Arsenophonus*, *Cardinium* and *Fritschea*) using genus-specific primers targeting the 16 S or 23 S rDNA genes was performed as previously described by Skaljac *et al*. [26–28]. PCR products were extracted from the gel and sequenced (Hylabs, Rehovot, Israel). Sequences were initially analyzed using BLASTn (www.ncbi.nlm.nih).

### Datasets and sequence analyses

*B*. *tabaci* mtCOI sequences available in GenBank (www.ncbi.nlm.nih.gov/) published until January 2017 with their geographical source, were retrieved. A total of 4,253 sequences from 82 countries were identified; 2,903 accessions were selected because they contained the 657 bp fragment of the 3’ end of mtCOI, required for further analysis. Clustal W algorithm was used to align the sequences and the identities were analyzed using BioEdit v7.0.9.0 (www.mbio.ncsu.edu/BioEdit), followed by visual inspection and manual adjustment. The sequences were translated into their corresponding amino acids to check for correct reading frame. To confirm the geographically distributed *B*. *tabaci* species, we compared the sequences with the 42 putative species sequences already published [7,10–14]. All 193 representative *B*. *tabaci* species from the different countries were recovered from GenBank sequences and are detailed in S1 Table.

All *B*. *tabaci* endosymbionts sequenced as part of this study, along with geographically available in the database (www.ncbi.nlm.nih.gov/). A total of 1,226 sequences were present in the database from 32 countries, of these 298 accessions were selected based on the sequences that containing alignment overlapping regions. For the purpose of detailed analysis, sequences from *Bemisia* genetic groups only considered for further analysis. Endosymbiont sequences were aligned using Clustal W algorithm to align the sequences and their identities using BioEdit v7.0.9.0 (www.mbio.ncsu.edu/BioEdit), followed by manual removing of sequences.

### Phylogenetic analyses of *B*. *tabaci* and endosymbionts

To construct a comprehensive *B*. *tabaci* phylogenetic tree, we first collected mtCOI sequences from each country and conducted neighbor-joining tree with MEGA 6.0 [54]. Duplicated species were excluded; only representative sequences of each species from each country were included. Phylogenetic trees were constructed based on the 192-nucleotide mtCOI sequences of *B*. *tabaci* species and *B*. *afer*, *B*. *atriplex*, *B*. *subdecipiens*, *Trialeurodes vaparariorum*, *T*. *abutilonea*, *T*. *ricini*, *T*. *lauri* and *Tetraleurodes acacia* as outgroups. Genetic divergences were calculated from the 52 mtCOI sequences using P-distance and Kimura 2-parameter (K2P) distance models in MEGA 6.0 [54].

Multiple alignments of the mtCOI and 16S/23S rRNA sequences were generated using the program MAFFT 7 [55]. The alignments were then inspected and corrected manually. The appropriate model of evolution was estimated with Bayesian Information Criterion (BIC) with Jmodeltest 2.1.6 on CIPRES Science Gateway [56] the selected models were the same as for maximum likelihood inferences. The models selected were GTR+G for mtCOI, JC+G for *Porteira* (16S), HKY for *Wolbachia* (16S), GTR for *Cardinium* (16S), GTR+I for *Rickettsia* (16S) and *Hamiltonella* (16S), HKY+G for *Arsenophonus* (23S). Using these models phylogenetic reconstruction was carried out using Bayesian inference approach (BI). For BI analysis, two independent runs of Markov chain Monte Carlo (MCMC) were run for 10 million generations using eight chains and sampling frequency of 1000 generations by MrBayes ver. 3.2 [57]. The first 25 per cent samples were discarded by setting burn-in fraction to 0.25. This burn-in setting was shared across the SUMT command to discard 25 per cent of sampled trees. Bayesian posterior probabilities were then calculated from the sample points after the MCMC algorithm began to converge. To ensure that our analyses are not trapped in local optima, four independent MCMC runs were performed. Topologies and posterior clade probabilities from different runs were compared for congruence. The consensus tree generated was visualized using FigTree available at http://tre.bio.ed.ac.uk/software/figtree.

## Results

### *B*. *tabaci* species identification

**Fig 1:**
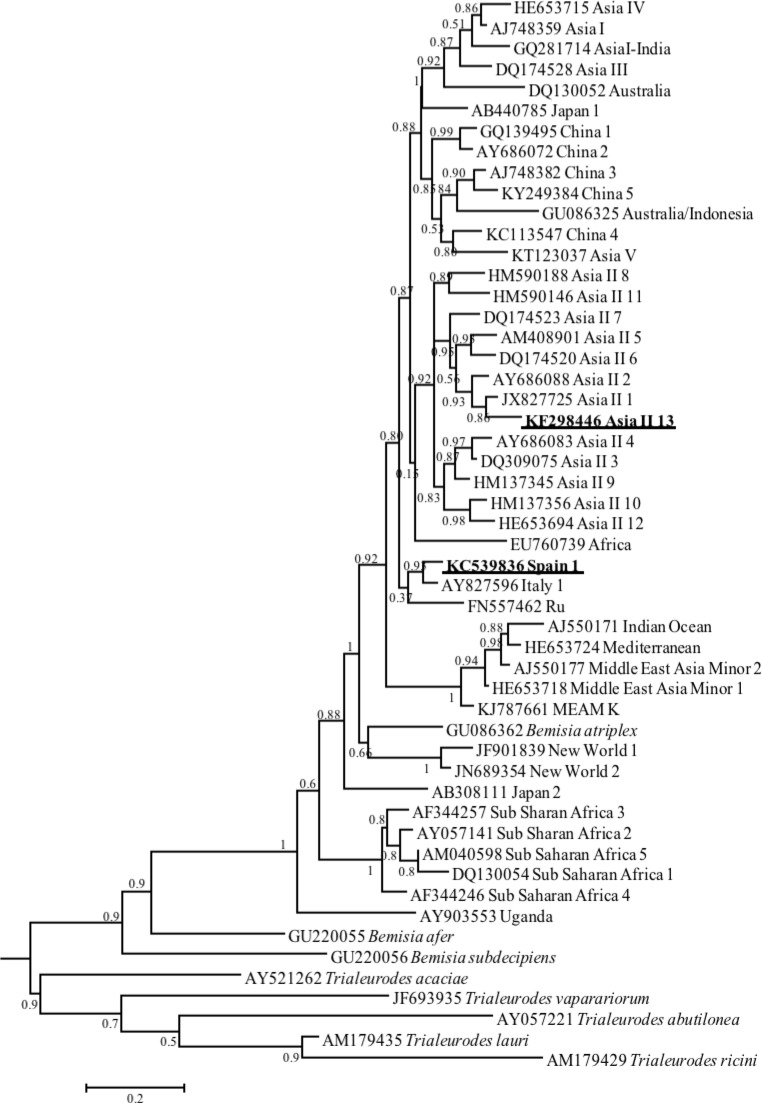
Bayesian phylogenetic tree inferred from mtCOI data using the GTR+G model. Posterior probabilities for the branches are given. The proposed new species are indicated in bold and underlined.

All together sixty whitefly populations collected in this study from Israel were identified initially based on Bem 23 F/R primers [53]. The same DNA was used to amplify the mtCOI sequences for determining the whitefly species. The phylogenetic analysis of mtCOI sequences revealed that 70% of the samples were *B*. *tabaci* MEAM1 while 30% were MED. Only representative sequences of MEAM1 and MED from Israel as well as representative endosymbiont sequences were included in the phylogenies generated afterwards for the worldwide analyses (Fig 1).

### mtCOI global *B*. *tabaci* sequence reanalysis and genetic diversity

To investigate the worldwide distribution of the putative *B*. *tabaci* species, 2,903 sequences were selected from 82 countries. Asia, Africa, North and South America, Europe and Australia were represented. Next, phylogenetic analyses were employed to infer the geographical distribution of *B*. *tabaci* putative species. Two sequences (accessions KC539836 from Spain and KF298446 from India) were separated from the other 42 species in the phylogenetic tree (Fig 1). The comparison conducted with the 42 *B*. *tabaci* putative species, showed that the above two sequences have pairwise divergences that exceed 4%, either with the consensus sequences or between themselves suggesting that they represent new species. KC539836 was clustered with Italy 1 (5%); and it was named Spain 1 in this study. KF298446 from India clustered with Asia II 1 (7%) species; and it was named Asia II 13 in this study. Therefore, two putative species, Asia II 13 and Spain 1, were identified and added to the previously reported 42 groups. The reclassification based on the 3.5 and 4% genetic divergence revealed that the 44 groups are clearly distinct (Fig 1, S2 Table, S1 Fig).

### Continental separation of *B*. *tabaci* populations

**Fig 2:**
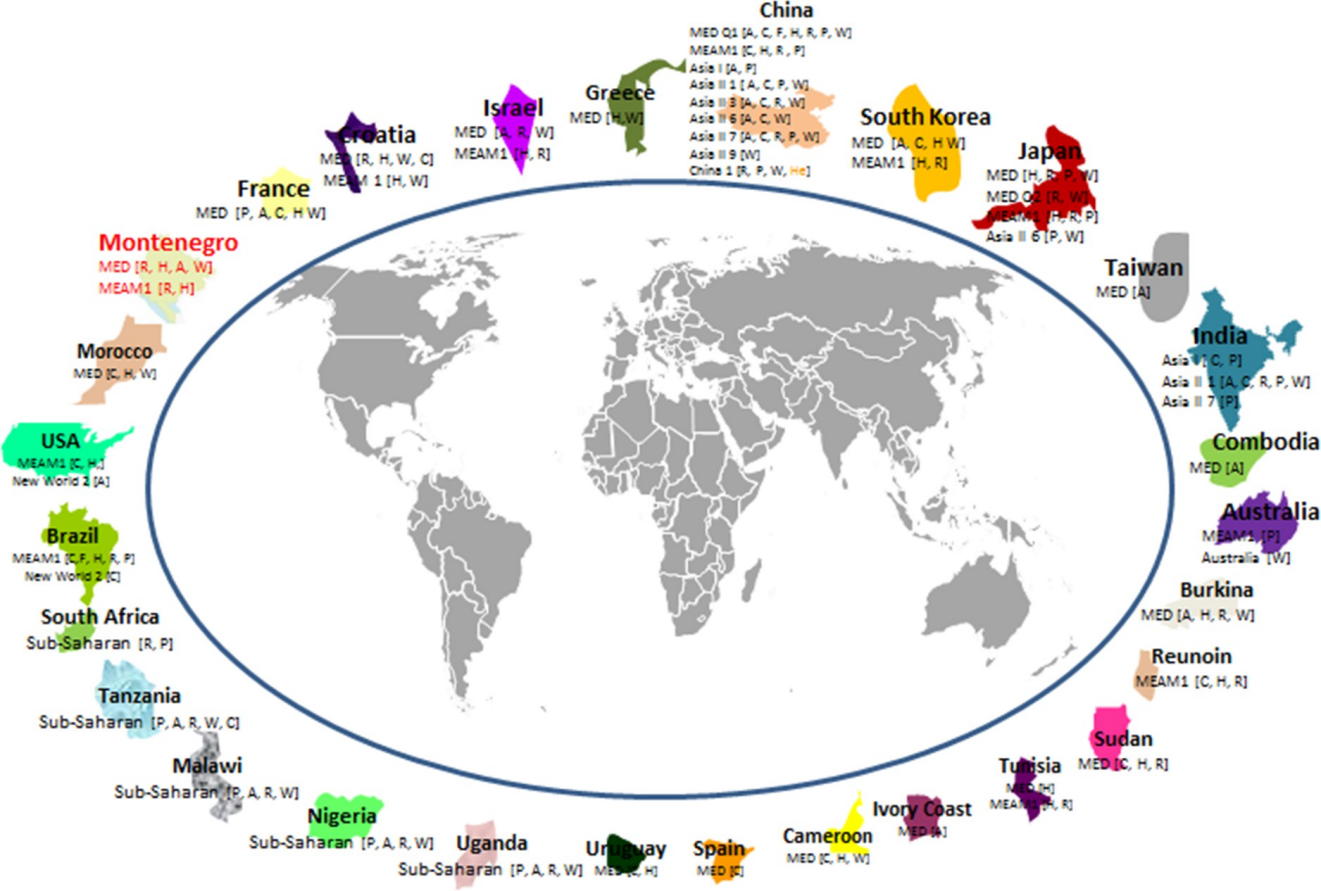
Schematic worldwide geographical distribution of *Bemisia tabaci* cryptic species and its bacterial endosymbionts. *B*. *tabaci* endosymbionts in square brackets: ***A****-Arsenophonus*, **C**-*Cardinium*, **F**-*Fritschea*, **H**-*Hamiltonella*, **R**-*Rickettsia*, **P**-*Porteira*, **W**-*Wolbachia*, ***He****-Hemipteriphilus*.

The phylogenetic analyses revealed that the Asian continent contains the highest number of *B*. *tabaci* species, with a total of 28 native and invasive species (Asia I, Asia I-India, Asia II 1–13, Asia III, Asia IV, MED, MEAM1, MEAM K, China 1–4, Australia/Indonesia, India, Japan 1–2) distributed across 13 countries (India, Pakistan, Bangladesh, Nepal, Cambodia, Indonesia, Malaysia, Myanmar, Singapore, China, Japan, South Korea and Taiwan). In addition to the MEAM1 (B biotype), the results demonstrated the geographic distribution of MED (Q biotype) across the Middle East (Egypt, Israel, Syria and Turkey), South East Asia (Cambodia and Malaysia), East Asia (China, Japan, South Korea and Taiwan), Africa (Algeria, Benin, Burkina Faso, Cameroon, Ghana, Ivory Coast, Morocco, Nigeria, Senegal, Sudan, South Africa, Tanzania, Togo, Tunisia, Uganda, Zimbabwe and Reunion Island), Europe (Bosnia Herzegovina, Croatia, Cyprus, Czech Republic, France, Greece, Italy, Netherlands, Portugal, Spain), North America (Guatemala, USA, Canada and Mexico) and South America (Argentina, Brazil and Uruguay). Furthermore, the putative Sub-Saharan African 1–5 species and the Indian Ocean were restricted to the Mediterranean area and Africa. The New World species which are native to the Americas were also detected in the Old World (Sudan). Altogether, MEAM1 was reported in 42 countries while MED from 44 countries, showing that these species are the most highly diverse and distributed on a worldwide scale. The species that are endemic to China were described in 4 countries, the Asian species in 14 countries, the Indian Ocean species in 6 countries, Sub-Saharan Africa in 19 countries and the New World species in 12 countries. The newly described species Asia I-India, Asia II 2, Asia II 3, Asia II 4, Asia II 8, Asia II 9, Asia II 10, Asia II 11, Asia II 12, Asia II 13, Asia IV, Asia V, Australia, Australia/Indonesia, China 4, China 5, MEAM K, Italy 1, Japan 1, Sub Saharan Africa 5 and Uganda, each was described in only one country (Table 1, Fig 2).

**Table 1:** Worldwide distribution of *Bemisia tabaci* species.

No.	Country	*B*. *tabaci* species
	**Old World**	
	**South Asia**	
1.	India	Asia I, Asia I-India, Asia II 1, Asia II 5, Asia II 7, Asia II 8, Asia II 11, Asia II 13, MEAM K, China 3, MEAM1
2.	Pakistan	Asia I, Asia II 1, Asia II 5, Asia II 7, MEAM1
3.	Bangladesh	Asia I, Asia II 1, Asia II 5, China 3
4.	Nepal	Asia II 1
	**South East Asia**	
5.	Cambodia	MED, Asia I
6.	Indonesia	Australia/Indonesia, Asia II 7, Asia II 12
7.	Malaysia	Asia I, MED, China 2, Asia II 7
8.	Myanmar	Asia II 5
9.	Singapore	Asia I
	**East Asia**	
10.	China	MED, MEAM1, Asia I, Asia II 1, Asia II 2, Asia II 3, Asia II 4, Asia II 6, Asia II 7, Asia II 9, Asia II 10, Asia IV, China 1, China 2, China 3, China 4
11.	Japan	MED, MEAM1, MEAM2, Asia I, Asia III, Japan 1, Japan 2, Asia II 1, Asia II 6
12.	South Korea	MED, MEAM1, Japan 2
13.	Taiwan	MED, MEAM1, Asia I, Asia III, Asia II 1, Asia II 6, Asia II 7
	**Middle East**	
14.	Egypt	MED, MEAM1
15.	Iran	MEAM1
16.	Iraq	MEAM1
17.	Israel	MED, MEAM1
18.	Jordan	MEAM1
19.	Kuwait	MEAM1
20.	Saudi Arabia	MEAM1
21.	Syria	Asia II 1, MED, MEAM1
22.	Turkey	MED, MEAM1, Asia I
23.	United Arab Emirates (UAE)	MEAM1
24.	Yemen	MEAM1
		
	**Africa**	
25.	Algeria	MED
26.	Benin	MED, Sub Saharan Africa 1
27.	Burkina Faso	MED
28.	Burundi	Sub Saharan Africa 1
29.	Cameroon	Africa, MED, Sub Saharan Africa 2, Sub Saharan Africa 3, Sub Saharan Africa 4
30.	Democratic Republic of the Congo	Sub Saharan Africa 1, Sub Saharan Africa 3
31.	Ghana	MED, Sub Saharan Africa 3, Sub Saharan Africa 1
32.	Ivory Coast	MED
33.	Kenya	Sub Saharan Africa 1, Sub Saharan Africa 2
34.	Madagascar	Indian Ocean
35.	Malawi	Sub-Saharan Africa 1
36.	Mali	Sub Saharan Africa 2
37.	Mauritius	Indian Ocean
38.	Morocco	MED, MEAM1, Spain 1
39.	Mozambique	Sub Saharan Africa 1
40.	Nigeria	MED, Sub Saharan Africa 2
41.	Senegal	MED, MEAM1
42.	Seychelles	Indian Ocean
43.	Sudan	MED, New World
44.	Swaziland	Sub-Saharan Africa 1
45.	South Africa	MED, MEAM1, Sub Saharan Africa 1
46.	Tanzania	MED, Sub Saharan Africa 1
47.	Togo	MED, Sub Saharan Africa 3
48.	Tunisia	MED, MEAM1, Sub Saharan Africa 2
49.	Uganda	Uganda, Indian Ocean, MED, Sub Saharan Africa 2, Sub Saharan Africa 5, Sub Saharan Africa 1
50.	Zambia	Sub Saharan Africa 1
51.	Zimbabwe	MED
52.	Mayotte	MEAM1
53.	Reunion	MED, MEAM1, MEAM2, Indian Ocean
	**Europe**	
54.	Bosnia and Herzegovina	MED
55.	Croatia	MED, MEAM1
56.	Cyprus	MED, MEAM1
57.	Czech Republic	MED
58.	France	MED, MEAM1, Indian Ocean, New World 1
59.	Greece	MED, MEAM1
60.	Italy	MED, MEAM1, Italy 1, Ru
61.	Netherlands	MED
62.	Netherlands Antilles	MEAM1
63.	Portugal	MED, Sub Saharan Africa 2
64.	Spain	MEAM1, MED, Sub Saharan Africa2, Sub Saharan Africa 3, Italy 2
65.	**Australia**	Australia, MEAM1
	**New World**	
	**South America**	
66.	Argentina	MEAM1, New World 2, MED
67.	Bolivia	New World 2
68.	Brazil	MEAM1,MED, New World 1, New World 2
69.	Colombia	New World 1
70.	Uruguay	MED
71.	Venezuela	New World 1, MEAM1
	**North America**	
72.	Cuba	MEAM1
73.	Belize	New World 1
74.	Trinidad and Tobago	MEAM1
75.	USA	MED, MEAM1, New World 1
76.	Canada	MED, MEAM1
	**Central America**	
77.	Honduras	New World 1
78.	Mexico	MED, MEAM1, New World 1
79.	Panama	New World 1
80.	Dominican Republic	MEAM1
81.	Guatemala	MED, MEAM1, New World 1
	**Micronesia, Oceania**	
82.	Nauru	Asia II 5
		
	**Greater Antilles, Caribbean**	
83.	Puerto Rico	MEAM1, New World 1

### Infection and co-infection with secondary bacterial endosymbionts

Individual whiteflies were analyzed for primary and secondary endosymbionts by 16S and 23S rRNA primers. As expected, bacterial infections were detected in both MEAM1 and MED species. MEAM1 populations from Israel showed infection with *P*. *aleyrodidarum*, *Rickettsia*, and *Hamiltonella*, while MED populations showed the presence of *P*. *aleyrodidarum*, *Rickettsia*, *Wolbachia* and *Arsenophonus*. *Cardinium* and *Fritschea* were not detected in any population tested. BLAST analysis of these 16S and 23S rRNA sequences confirmed infection with the respective endosymbionts in GenBank. All the sequences generated in this work were deposited in NCBI GenBank with accession numbers from KY620201 to KY620209.

### Worldwide distribution scenario of bacterial endosymbionts within the *B*. *tabaci* species

To investigate the worldwide endosymbiont diversity in *B*. *tabaci* species complex, we retrieved 16sRNA gene sequences of about 1,226 accessions of reported *B*. *tabaci* endosymbionts from GenBank. Of all these, a total of 298 sequences, including sequences from this study, were selected from 32 countries. All the six continents, Asia, Africa, North America, South America, Europe and Australia, were represented and provided an interesting geographical distribution and wide genetic diversity within the *B*. *tabaci* species complex. Based on the 16sRNA sequences available in the database, *P*. *aleyrodidarum* was reported from nine *Bemisia* cryptic species (MED, MEAM1, Asia I, Asia II 1, Asia II 6, Asia II 7, China 1, Japan, Sub-Saharan Africa), which were reported from fifteen countries, worldwide (China, Japan, France, Australia, Brazil, India, South Africa, Tanzania, Malawi, Uganda, Nigeria, USA, Mexico, Israel, Pakistan) (Fig 2). However, many *B*. *tabaci* populations around the world were studied without investigation of their *P*. *aleyrodidarum* sequences, thus the actual scenario of *P*. *aleyrodidarum* diversity might still be different from what is presented in this manuscript and we only relied our analysis on published sequences.

In case of the secondary symbionts, *Rickettsia* was associated with seven species (MED, MEAM1, Asia II 1, Asia II 3, Asia II 7, China 1 and Sub-Saharan Africa) in nineteen countries (China, Japan, Israel, Burkina, Montenegro, Croatia, South Korea, Sudan, Brazil, Tunisia, Reunion, Antilles, India, Bangladesh, Tanzania, Malawi, Uganda, Nigeria and Pakistan). *Cardinium* was associated with the species MED, MEAM1, Asia I, Asia II 1, Asia II 3, Asia II 6, Asia II 7, Japan, Sub-Sharan Africa and New World. *Arsenophonus* was associated with MED, Asia I, Asia II 1, Asia II 3, Asia II 6, Asia II 7, Sub-Sharan Africa and New World, while both *Fritschea* and *Hamiltonella* detected in individuals from MED, MEAM1 and New World 2. *Hemipteriphilus* was associated with China 1 species from China (Fig 2).

**Fig 3:**
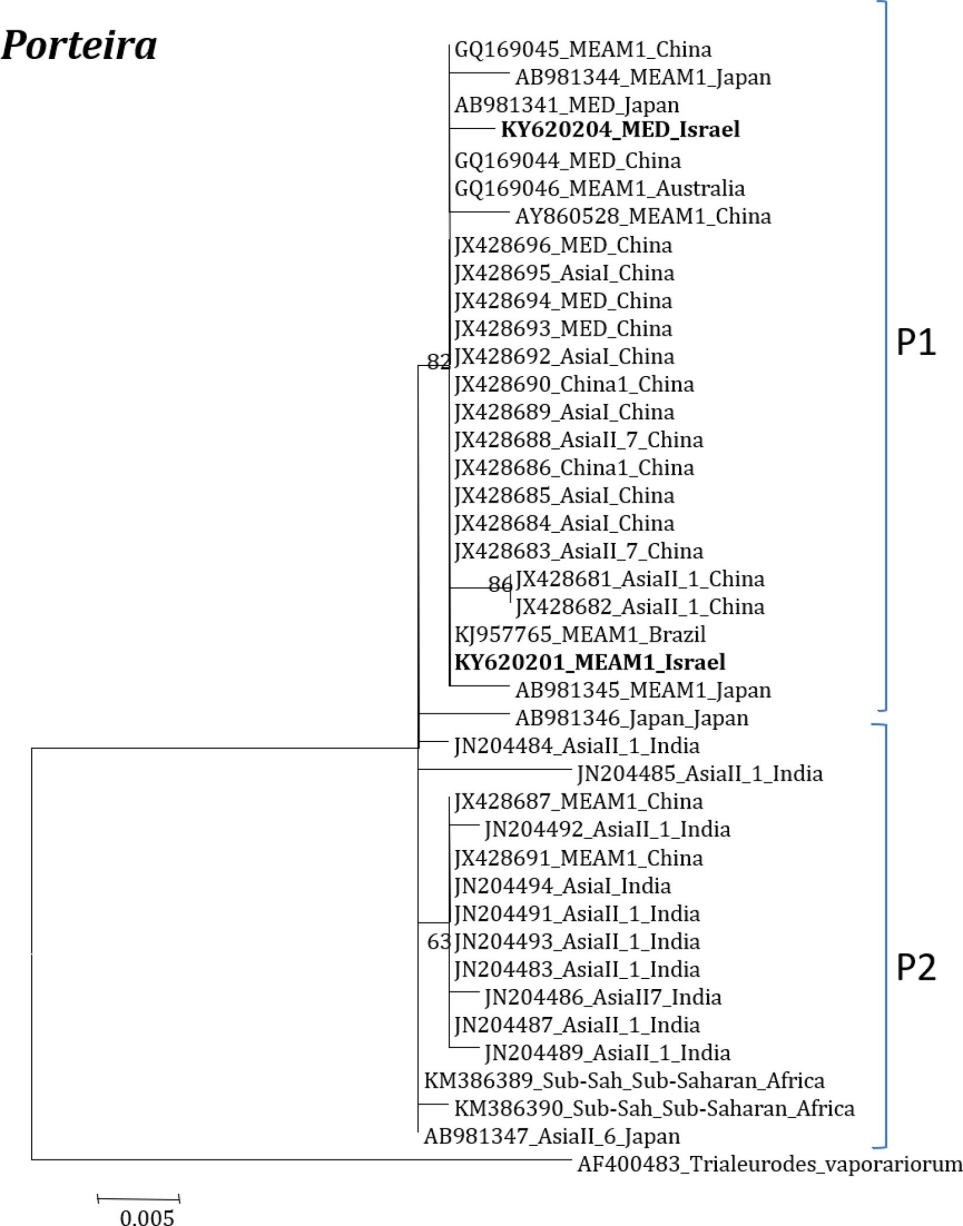
Molecular phylogenetic placements of *Porteira* (16S) from reported sequences of worldwide whitefly species. The tree was constructed via Bayesian inference (BI) using an JC+G substitution model. Sequences from Israel are indicated in bold.

**Fig 4:**
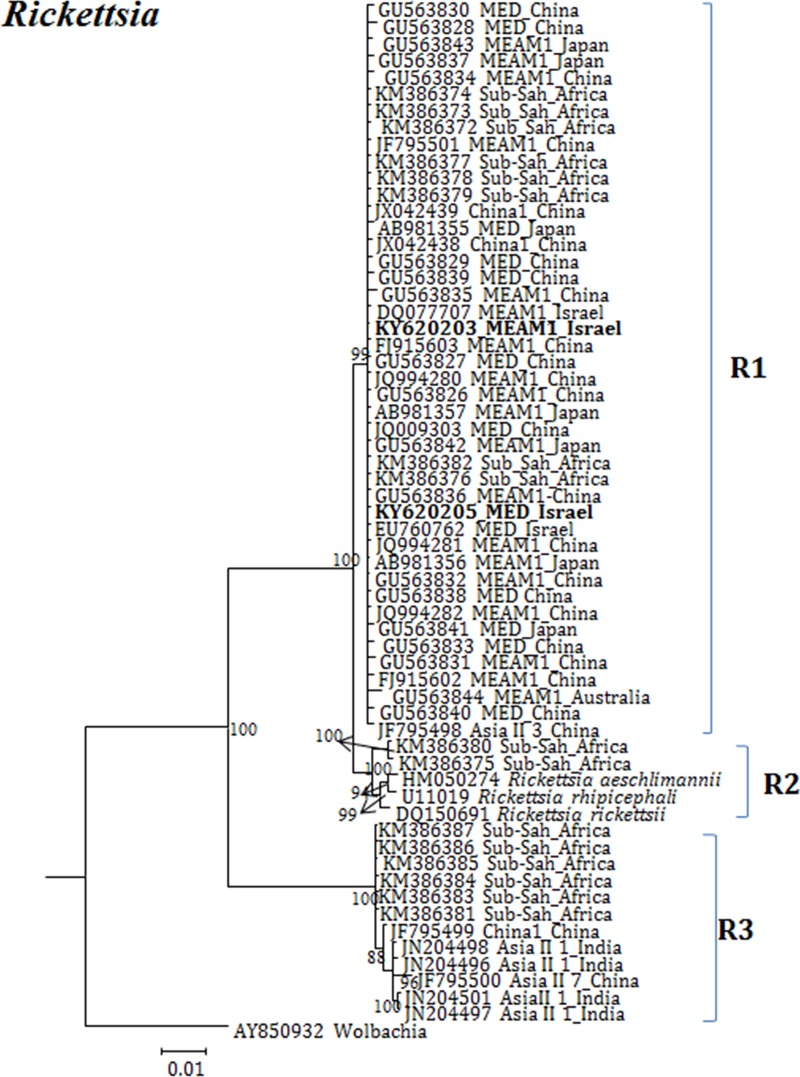
Molecular phylogenetic placements of *Rickettsia* (16S) from reported sequences of worldwide whitefly species. The tree was constructed via Bayesian inference (BI) using an GTR+I substitution model. Sequences from Israel are indicated in bold.

**Fig 5:**
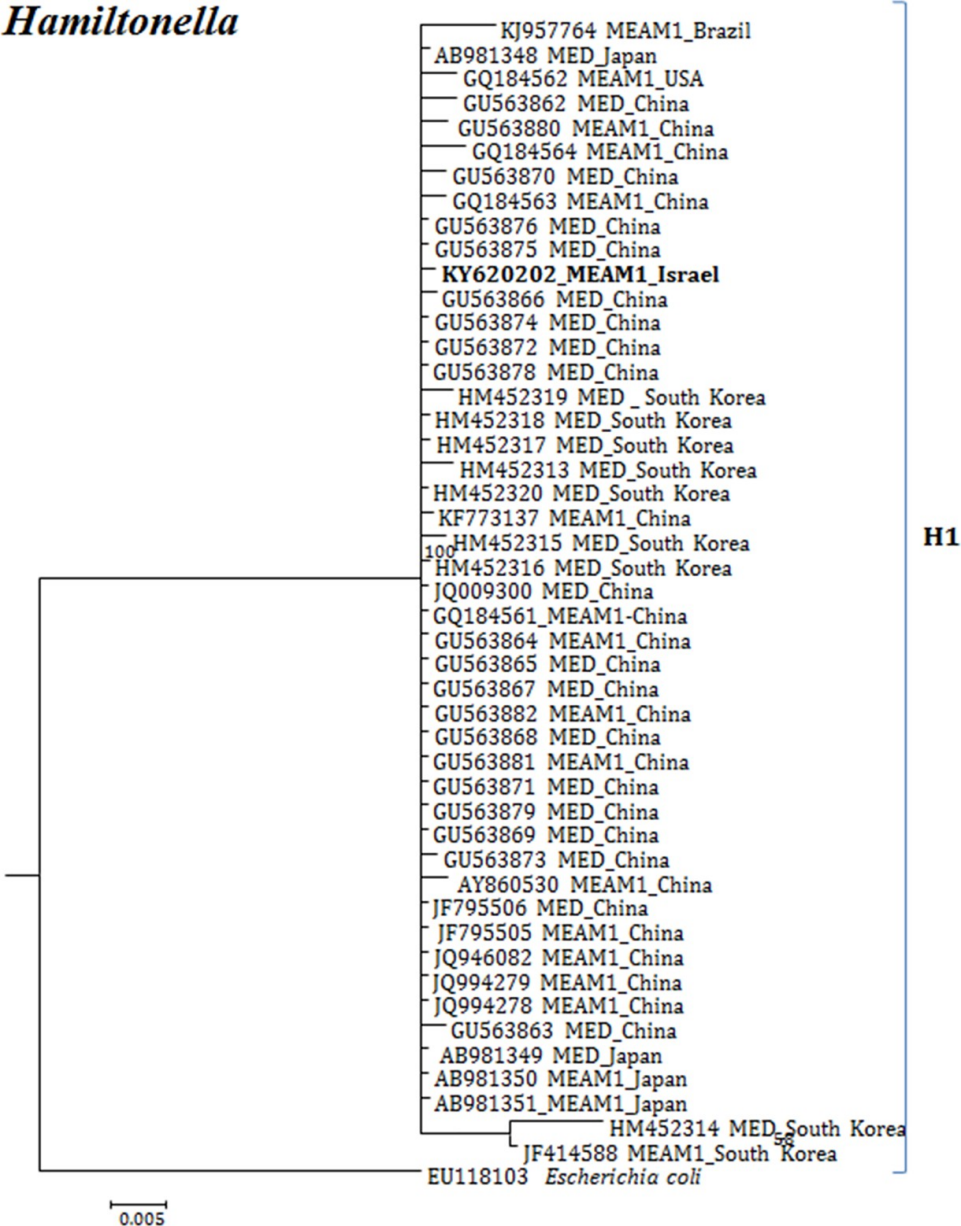
Molecular phylogenetic placements of *Hamiltonella* (16S) from reported sequences of worldwide whitefly species. The tree was constructed via Bayesian inference (BI) using an GTR+I substitution model. Sequences from Israel are indicated in bold.

**Fig 6:**
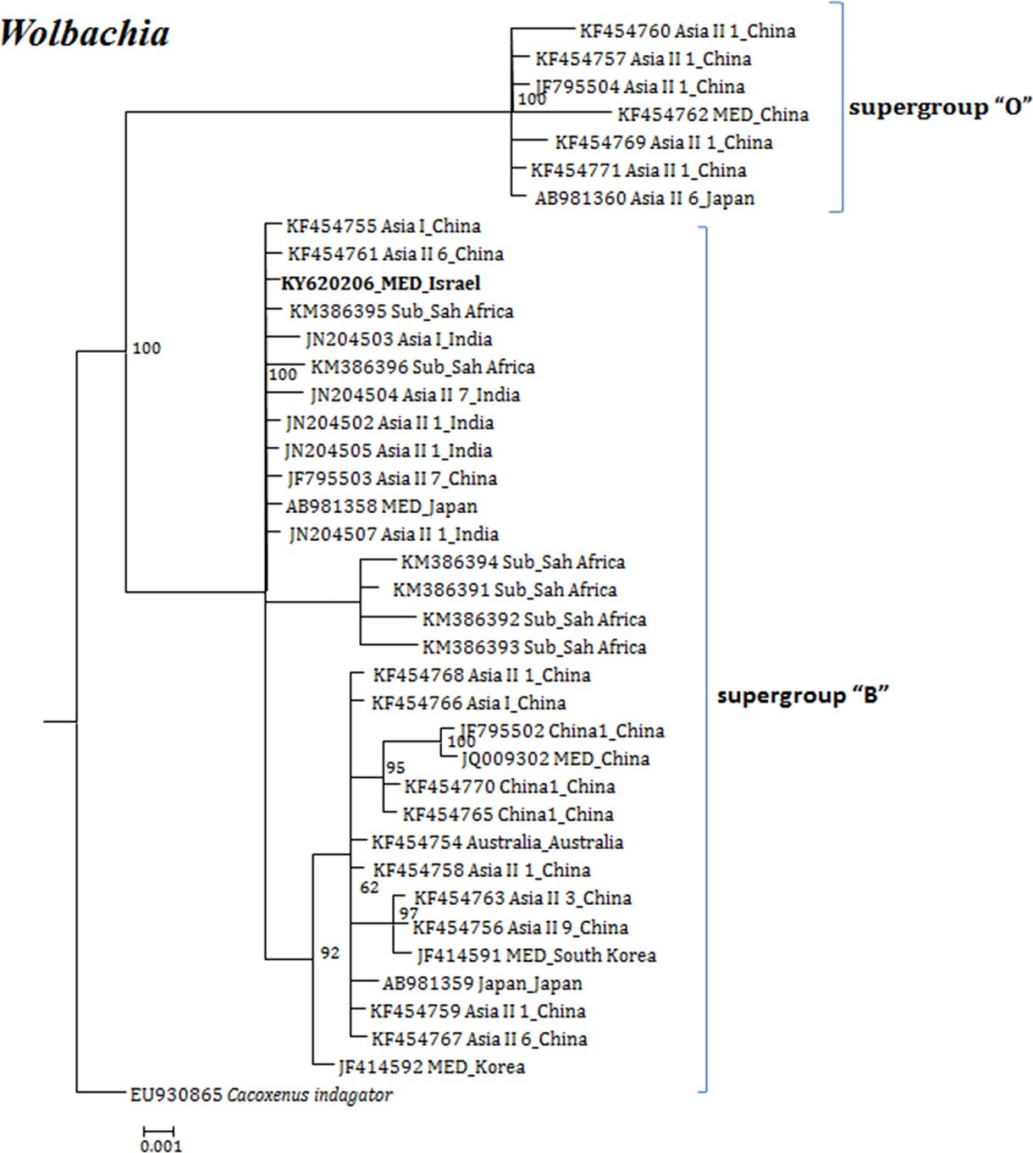
Molecular phylogenetic placements of *Wolbachia* (16S) from reported sequences of worldwide whitefly species. The tree was constructed via Bayesian inference (BI) using an HKY substitution model. Sequences from Israel are indicated in bold.

**Fig 7:**
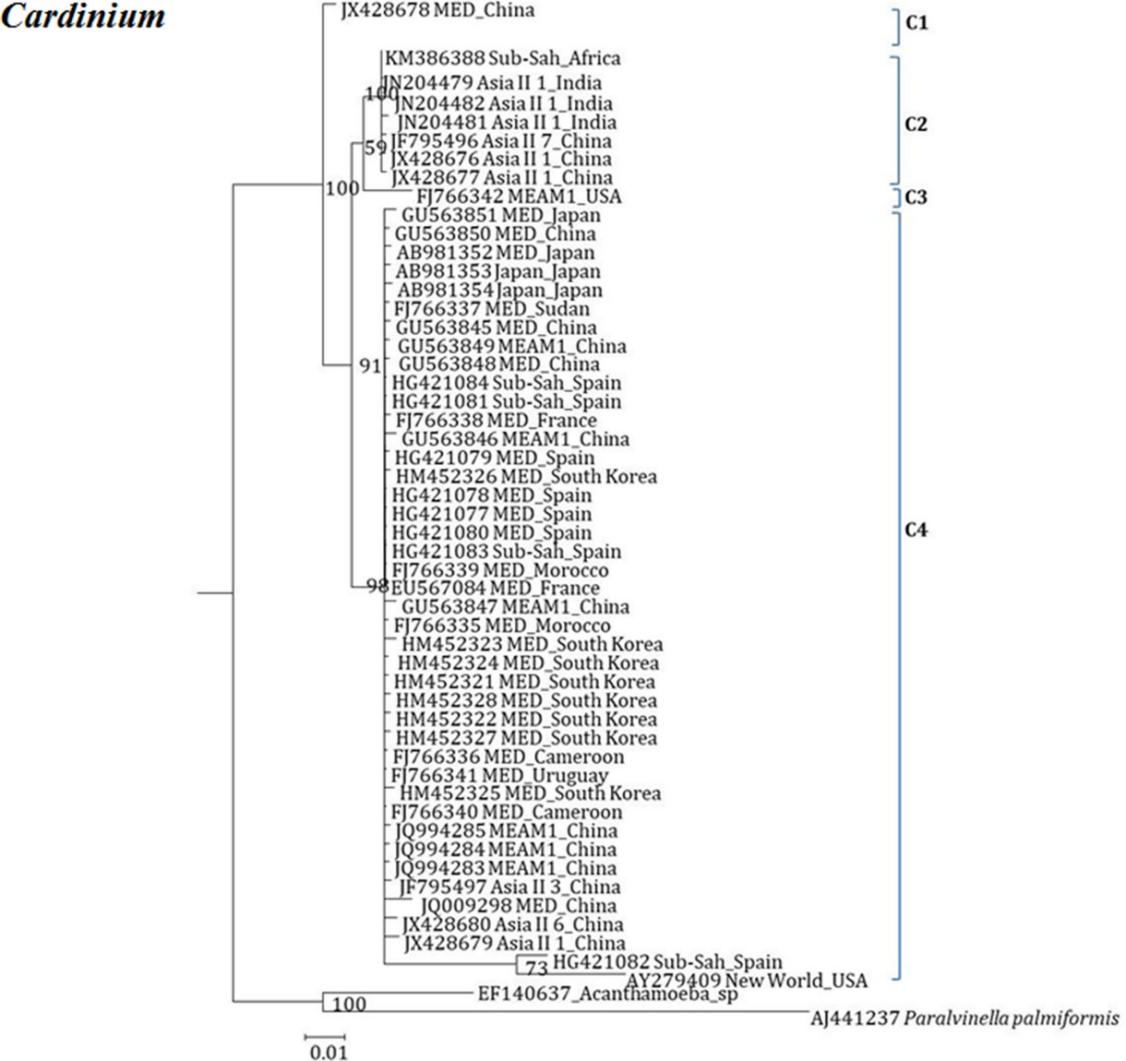
Molecular phylogenetic placements of *Cardinium* (16S) from reported sequences of worldwide whitefly species. The tree was constructed via Bayesian inference (BI) using an GTR substitution model. Sequences from Israel are indicated in bold.

**Fig 8:**
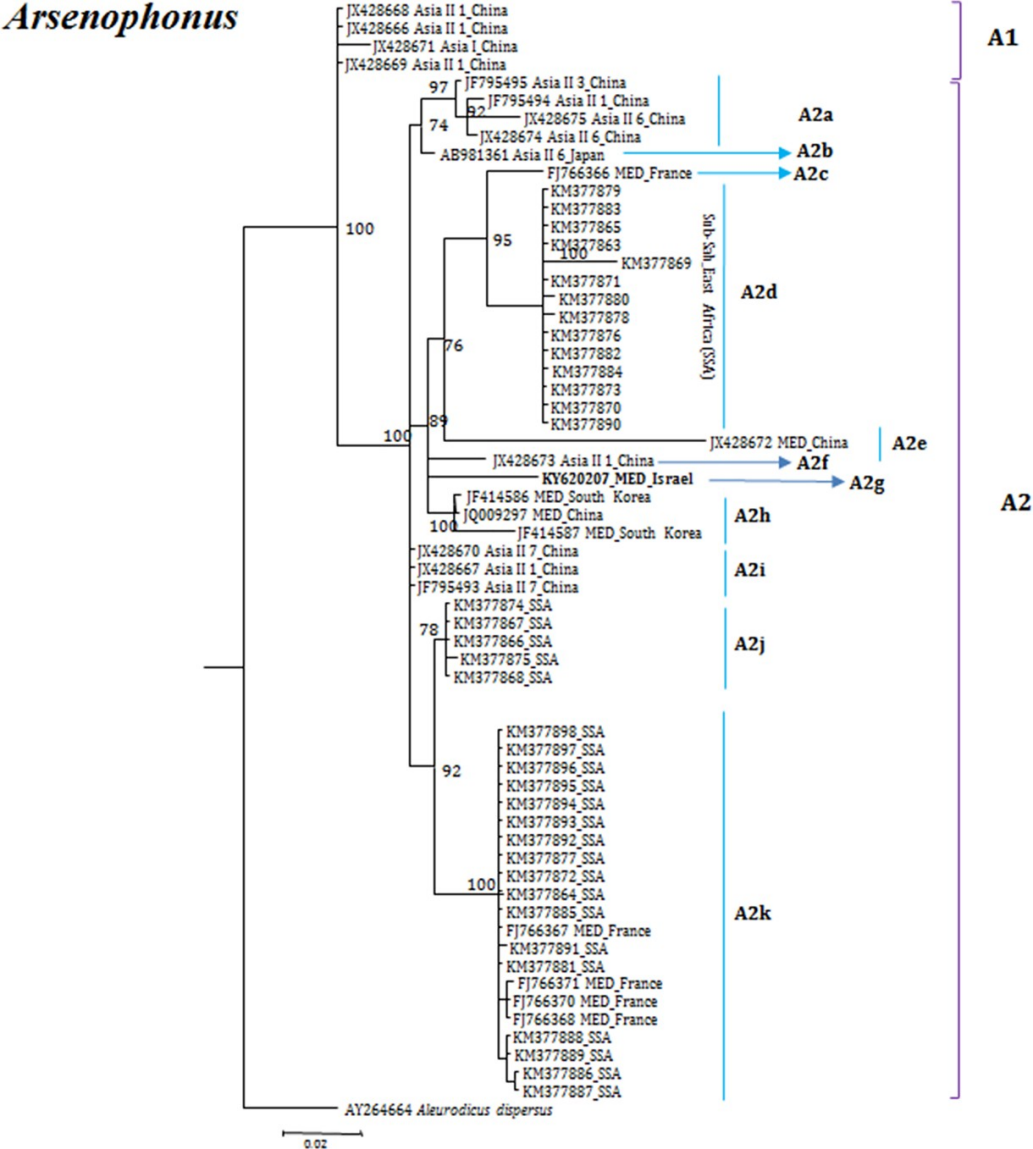
Molecular phylogenetic placements of *Aresenophonus* (23S) from reported sequences of worldwide whitefly species. The tree was constructed via Bayesian inference (BI) using an HKY+G substitution model. Sequences from Israel are indicated in bold.

Overall, we found evidence for *P*. *aleyrodidarum*, *Rickettsia*, *Wolbachia*, *Hamiltonella*, *Arsenophonus*, *Cardinium* and *Fritschea* association with MED. In case of MEAM1, except *Arsenophonus* and *Hemipteriphilus*, the remaining endosymbionts were associated with this species. Interestingly, *Fritschea* and *Hamiltonella* were restricted to MED, MEAM1 and New World species, and were not associated with *Bemisia* new complex species. Accession numbers of endosymbiont sequences obtained from the Gen Bank database are detailed in the respective phylogenetic trees (Figs 3,4,5,6,7 and 8).

### Phylogenetic analysis of the primary endosymbiont of *B*. *tabaci* cryptic species

The phylogenetic relationships among the endosymbionts were first estimated using the 16s rDNA sequences obtained from GenBank. The *P*. *aleyrodidarum* grouped into two major clades P1 and P2 with bootstrap scores of >70% (Fig 3). Both P1 and P2 includes both invasive and indigenous species from six countries. Interestingly, the percentage nucleotide identities of selected *Porteira* from each species from different countries showed a peculiar scenario; in which the bacterium from different species in India share >95% identity with species from Japan, Brazil and Sub-Saharan Africa, Australia, and *vice versa*. *Portiera* from China also shares <95% identity with *Portiera* reported from *B*. *tabaci* species of the above countries. *Porteira* in all species (includes both Indigenous and invasive species) that originated from China share the highest percentage similarities compared with other countries (S3 Table).

### Phylogenetic analysis of secondary endosymbionts of *B*. *tabaci* cryptic species

*Rickettsia* was grouped in to three major clusters, R1, R2 and R3. The top clade R1 (Fig 4) consists of *Rickettsia* from both invasive and indigenous members from Australia, China, Japan, Israel and Sub-Saharan African countries. Clade R2 consists of *Rickettsia* only from Sub-Saharan Africa species. Clade R3 consists of *Rickettsia* only from indigenous species that originated from Sub-Saharan Africa, Asia II 7 Asia II 1 from China and India.

Interestingly, published sequences for *Hamiltonella* from the invasive species (MED and MEAM1) all belonged to only one major cluster (H1) from Brazil, Japan, USA, China and South Korea (Fig 5).

In the case of *Wolbachia*, 16 super-groups are so far successively named A-Q and are infecting a wide range of arthropods and filarial nematodes [58]. In our analysis, two super-groups B and O were observed in the overall analysis of *B*. *tabaci Wolbachia* sequences. The O super-group was observed in MED and Asian species from China and Japan. The B super-group was found in both MED and MEAM1 invasive species from Asia, Sub-Sharan Africa, China 1, Japan and Australia, and in indigenous species. Interestingly, both super-groups were observed in MED from China (Fig 6).

The phylogenetic tree of *Cardinium* resulted in four major clusters (C1-C4). The C1 consists, invasive MED species from China; C2 consists, Asian, MEAM1, Sub-Sharan African species from 4 countries (India, china, Sub-Saharan Africa and USA). In case of C3, invasive MEAM1 species from USA and C4 consists MED, MEAM1, Japan, Sub-Saharan Africa, Asian and New World species from 10 countries (China, Japan, Sudan, Spain, South Korea, Morocco, France, Cameroon, Uruguay and USA) (Fig 7). Interestingly, isolates that belong to the three clades C1, C2 and C4 were present in China. The isolates that appeared in C4 included MED, MEAM1 and Asia II 1 and Asia II 6. Clade C2 consists only indigenous species (Asia II 1, Asia II 7 and Sub-Saharan Africa).

*Arsenophonus* phylogenetic analyses supported its grouping into two major groups (A1 and A2), the top clade A1 consisted only indigenous Asian species from China. Interestingly, the second clade A2 consists eleven subclades named as A2 (a-k). In these clades, Chinese *Arsenophonus* strains from invasive species observed in the two subclades A2e and A2h and indigenous species in three subclades A2a, A2f and A2i. Similarly, strains from Sub-Sahran Africa were more distinct and diverse within the Sub-Saharan Africa species. In this analysis, all isolates from Sub-Saharan Africa were observed only in the major clade A2, in which they sub-grouped into three clades A2d, A2j and A2k (Fig 8).

The overall analysis for all endosymbionts showed that, except *Hamiltonella* (Fig 5), the rest of the secondary endosymbionts were more diverse and appeared in different genetic groups in invasive and indigenous *B*. *tabaci* species than previously assumed. Interestingly, *Hamiltonella* was observed in both invasive species (MEAM1 and MED species worldwide) and in the New World 2 species from Brazil. In contrast, *Fritschea* was only observed in MED, MEAM1 and New World 2 species while *Hemipteriphilus* was detected in China1 species from China.

## Discussion

The whitefly *B*. *tabaci* species complex constitutes the most economically and agriculturally important insect pest worldwide. This pest is considered a super-vector and possibly is the most important insect vector transmitting viruses in agricultural crops worldwide [59,60]. It transmits more than 100 plant viruses and those are considered the most important in terms of damage they cause on a worldwide scale. This complex of cryptic species or biotypes differ in their behavior, plant host adaptations, ability to develop resistance to pesticides and induce plant disorders, ability to transmit plant viruses, the bacterial endosymbionts they harbor [26–28], and their genetic make-up, as has recently been described in the genomes of the two most widespread members of this complex: MEAM1 and MED species [61,62]. Most importantly, *B*. *tabaci* is rated among the 100 most invasive species worldwide. One of the most studied aspects of this complex, is the bacterial endosymbionts that infect members of this complex. Those bacteria were shown to greatly impact many aspects of the biology of *B*. *tabaci* including the ability to vector plant viruses, which in turn have significant impact on the damage caused by members of this species complex, especially the MEAM1 and MED species [63,64]. We thus attempted to collect all published sequences of bacterial symbionts from populations reported around the world and analyze their relationships. These analyses might shed light of the origins and relationships between different *B*. *tabaci* species and populations within and between countries and continents.

Several recent studies have suggested that biological and molecular differences between members of *B*. *tabaci* complex of cryptic species or biotypes warrants further analyses to clarify whether those members can be considered different species. Global phylogenetic reconstruction of the *B*. *tabaci* complex species was conducted with 366 sequences and around 12 major genetic groups were generated [12]. Later, De Barro *et al*. [11] and Dinsdale *et al*. [7] suggested that the term "cryptic species" be used to distinguish the whitefly population based on 3.5% mitochondrial cytochrome oxidase gene sequence divergence. Based on this criterion, 24 *B*. *tabaci* cryptic species were determined and nominated. Recently, Lee *et al*. [15] observed that a 4.0% genetic boundary was more realistic than 3.5% in distinguishing the *B*. *tabaci* species complex members. Though few controversies did occur [65,66], reclassification based on the 3.5% and 4% genetic divergence revealed that 42 groups are clearly distinct (Fig 1).

The newly described morphologically indistinguishable species (which include Africa, Asia I, Asia I-India, Asia II 1–12, Asia III, Asia IV, Asia V, Australia, Australia/Indonesia, China 1–5, Indian Ocean, Ru, Middle East Asia Minor I-II (MEAM), Mediterranean (MED), MEAM K, New World 1–2, Japan 1–2, Uganda, Italy 1, and Sub Saharan Africa 1–5) have been currently delimited at the global level [7,10–14]. The worldwide *B*. *tabaci* mtCOI sequence analysis that we conducted in this study supports the existence of two new *B*. *tabaci* species: Asia II 13 and Spain 1. This is the first report that shows the existence of 44 *B*. *tabaci* species worldwide.

Our analyses focused on comprehensively studying and analyzing the bacterial endosymbiont communities that infect wide geographic range of the *B*. *tabaci* species complex members, undertaken to gain more thorough understanding of endosymbiont diversity and complexity. Our results revealed complex divergence of the different endosymbiont sequences analyzed, except for *Hamiltonella*, which showed only one genetic group across the different worldwide populations analyzed. Two *Porteria* groups were identified in this analysis: P1 and P2. The sequences belonging to the P1 group were present in Australia, Brazil, China and Japan, whereas P2 group was identified in China, India, Japan and Sub-Sharan African populations. Similarly, total of four groups of *Cardinium* (C1-C4) were identified, in which C1 from China, C2- China, India and Sub-Saharan Africa, C3- USA, Cameroon, China, France, Japan, South Korea, Spain, Sudan and Uruguay, while the C4 was also widespread in a worldwide scale. *Wolbachia* super-groups O and B were identified. The B group members were identified in Australia, China, Japan, India, Sub-Saharan Africa and South Korea, while the O members in China and Japan. Other groups of *Wolbachia* that were reported from other arthropods were not identified in our study, suggesting that these two groups were acquired in earlier events of *B*. *tabaci* speciation and coevolved with the different members of the insect species complex, as they appear in many members of the group.

An important observation that could be drawn from our analysis that although more than 40 cryptic species of *B*. *tabaci* are identified, endosymbionts which infect phylogenetically remote members of this complex are sometimes grouped in the same clades. This applies to almost all symbionts except for *Hamiltonella* which showed only one group infecting all reported species. This observation, combined with the fact that evidence for direct horizontal transmission of the majority of these symbionts does not exist, although some evidence has been shown [67], suggests that the majority of the symbionts were acquired before the start of *B*. *tabaci* complex speciation from other whitefly species. Thus, their spread in whitefly populations, including *B*. *tabaci* species, occurred after this speciation. Their co-evolution with *B*. *tabaci* species and the intimate symbiotic associations they have developed with the whitefly also limited the evolved variations in their genomes. This observation further supports the fact that those symbionts are unevenly distributed between the different species complex members as shown in previous studies [26–29,40]. This is evident from the variation observed in the infection of the different symbionts in the different species complex members, and that not all reported symbionts infect all *B*. *tabaci* species complex members. The fact that although many of these members share many of the host plants and yet are significantly different in their symbiotic complements suggest that horizontal transmission via plants in indeed unlikely, as has been shown for *Rickettsia* [36,40].

It is known among *B*. *tabaci* endosymbionts, but also among other arthropods, that mixed infections with more than one symbiont exist, and *B*. *tabaci* harbors the highest number of mixed infection in one insect [28,29]. The different endosymbionts presented in this paper were reported to coexist in *B*. *tabaci* populations, sometimes inside the same organ [29,33]. This coexistence however, is also sometimes the cause for the uneven distribution of these symbionts in *B*. *tabaci* populations, as it is the cause for competition for space and resources in the insect by these symbionts, and the cause for immune system responses in the insect for maintaining levels of the symbionts inside the insect that do not interfere with its proper physiology and development. Although there is some chance that multiple horizontal events may be the cause for mixed infection, as mentioned above, the ability of horizontal transfer was only reported for *Rickettsia*. It thus remains more likely that early acquisition events of these symbionts is the cause for the diversity observed in the *B*. *tabaci* species complex, combined with the low probability that some of these symbionts could be distributed via horizontal transfers. Horizontal transfer could also occur between different species of whiteflies for example *B*. *tabaci* and *T*. *vaporariurom*, which also share the same host plants and have been shown to be infected with the same endosymbiont species [26–28].

In summary, we have performed combined analyses for the largest available datasets of both *B*. *tabaci* species and its endosymbiont sequences which resulted in a global diversity and geographic distribution of this important insect pest and its associated bacterial endosymbionts. These results will be helpful in understanding the successful invasions of *B*. *tabaci* species complex members across the globe, and the possible contribution of their associated symbionts in their invasions and abilities to cause damage in agricultural ecosystems.

## Supporting information

S1 Table Details of origin, species group, accession number and mtCOI sequences of 193 individuals of *B*. *tabaci* of species complex. (XLSX)

S2 Table Intraspecific generic divergences between species of the *B*. *tabaci* complex. (XLSX)

S3 Table Percentage nucleotide identities of selected *Porteira* from each species from different countries. (XLSX)

S1 Fig A Phylogenetic tree reconstructed based on mtCOI sequences of host *B*. *tabaci* using Bayesian analysis under GTR+G model. The bootstrap values are indicated. More Asia II 1, Spain 1 and Italy 1 sequences were included.
(PDF)
