# Supplementary figures and images for: Global genetic diversity and geographical distribution of Bemisia tabaci and its bacterial endosymbionts
Source: PLoS One. 2019 Mar 19;14(3):e0213946. doi: 10.1371/journal.pone.0213946 (PMC6424426; doi:10.1371/journal.pone.0213946)

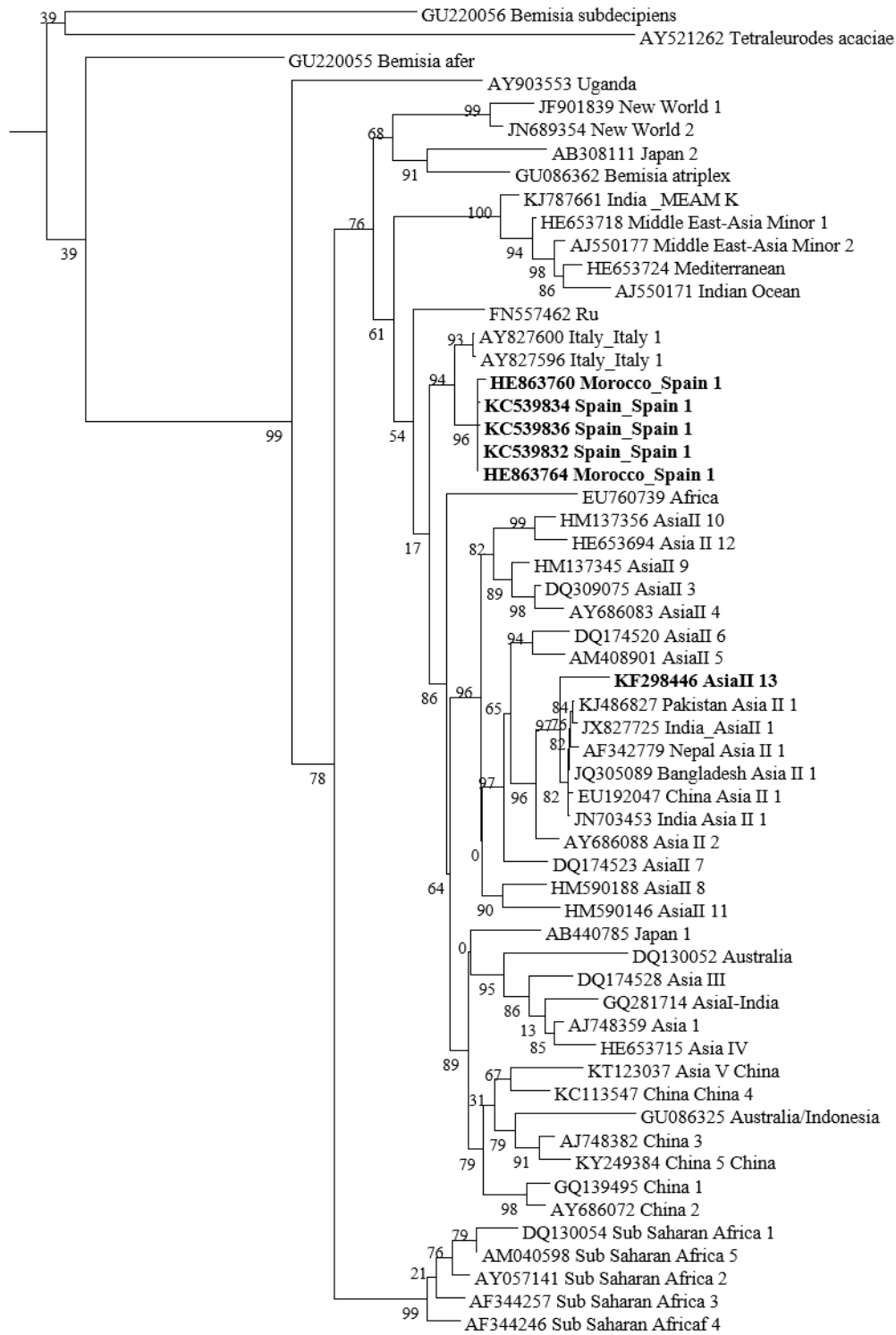

0.1

Supplement: S1 Fig — The bootstrap values are indicated. More Asia II 1, Spain 1 and Italy 1 sequences were included. (PDF) [file pone.0213946.s004.pdf]
